# Editorial: Mechanisms of prokaryotic predation, volume II

**DOI:** 10.3389/fmicb.2023.1256252

**Published:** 2023-07-27

**Authors:** David E. Whitworth, Juana Pérez, Robert J. Mitchell, Samay Pande

**Affiliations:** ^1^Department of Life Sciences, Aberystwyth University, Aberystwyth, United Kingdom; ^2^Department of Microbiology, Faculty of Science, University of Granada, Granada, Spain; ^3^Department of Biological Sciences, Ulsan National Institute of Science and Technology, Ulsan, Republic of Korea; ^4^Department of Microbiology and Cell Biology, Indian Institute of Science, Bengaluru, India

**Keywords:** predator, prey, myxobacteria, *Bdellovibrio*, modeling, transcriptomics

Bacterial predator-prey interactions are able to shape microbial communities and can profoundly influence the abundance and diversity of other microorganisms, including those with biotechnological or agricultural applications. The weapons and strategies used by these predators to kill and consume prey, and the adaptations and/or defensive mechanisms that the prey use to respond and adapt to the biotic stress caused by the predator, are complex and vary depending on the bacterial species involved. This Research Topic has brought together two research articles and three reviews, which collectively highlight the importance and potential applications of understanding the mechanisms of prokaryotic predation.

Two complementary studies have provided insights into this field by describing the transcriptomic changes that a predator undergoes in the presence of its prey (predatosome) and the whole set of genes in the prey that alter their transcription in response to the predator (defensome). Those transcriptional changes, which might reflect the mechanisms used to attack prey, resist predation or compete for resources, have been deciphered during the early stages of the interaction between *Myxococcus xanthus* and *Sinorhizobium meliloti*. *M.xanthus* is a soil bacterium (sharing its habitat with many other soil bacteria, including the nitrogen-fixing legume symbiont *S.meliloti*). It has been extensively studied because of its unique multicellular lifecycle, that includes a facultative epibiotic predatory stage and a developmental stage (in the absence of nutrients) that culminates with the formation of fruiting bodies filled with resistant cells called myxospores.

Pérez et al. found that the *M. xanthus* predatosome includes the upregulation of many genes encoding hydrolytic enzymes and proteins involved in the biosynthesis of secondary metabolites, that will help the cells to detect, kill, lyse, and consume prey, but they also found that *M. xanthus* modifies its lipid composition during predation. Additionally, during predatory attack, many regulatory elements decrease their expression levels and comparison with the developmental transcriptome reveals that a significant number of those downregulated genes are essential for development.

In contrast, Soto et al. discovered that the defensome includes the up-regulation of genes involved in protein synthesis and secretion, energy generation, and fatty acid (FA) synthesis, and the down-regulation of genes required for FA degradation and carbohydrate transport and metabolism. The results obtained also suggest that a remarkable remodeling of the cell envelope, transport of peptides and production of H_2_O_2_ and formaldehyde are important players in the response of *S. meliloti* to *M. xanthus* predation. Furthermore, induction of the iron-uptake machinery in both the predator and the prey indicates strong competition for this metal during predation.

The predatory myxobacteria are also the subject of the mini-review of Phillips et al., who highlight a variety of research challenges facing myxobacteriologists. Comparative genomics has allowed objective classification of myxobacterial isolates, and an appreciation of their biosynthetic gene cluster (BGC) diversity. BGCs direct the production of specialized secondary metabolites, which are rapidly disseminated by horizontal gene transfer. Phillips et al. challenge the traditional assumption that a strain's predatory activity is a consequence of its BGCs, highlighting their rapid gain/loss from individual strains, and alternative non-predatory functions of secondary metabolites. They also call for harmonization of methods between laboratories, further studies linking experimental observations with ecology and for an increased focus on ecologically relevant myxobacteria rather than model organisms.

Two reviews in this Research Topic focus on the predatory mechanisms of the endobiotic predator *Bdellovibrio bacteriovorus*, which attaches to Gram-negative prey cells and invades the prey periplasm, forming a bdelloplast. The predator then consumes the prey cell contents, reproducing within the bdelloplast before lysing it and releasing progeny cells which swim off to hunt for more prey.

Lai et al. provide a historical account of 60 years of research into *B. bacteriovorus* and provide a state-of-the-art summary of what is known about each stage of the predatory life-cycle. The authors highlight how researchers have repeatedly applied new technologies to leverage novel insights into *B. bacteriovorus* predation, and prioritize five key questions for future studies. Some questions have particular relevance to *B. bacteriovorus*, addressing the mechanisms of genome compaction, the trigger for concluding growth, and the exit of progeny cells from the bdelloplast. Others topics have broader relevance to studies of all bacterial predators, including defining what dictates prey specificity, and how the predator accesses the contents of the prey cytoplasm.

Mathematical models of predator-prey dynamics have been used for around a century, but their application to prokaryotic predation has been limited. In their review, Summers and Kreft focus on the use of mathematical modeling to provide biological insights into predation by *B. bacteriovorus*. They explain how mathematical modeling has allowed testable predictions to be made regarding predation dynamics, and how modeling can play a role in developing strategies for the effective use of *B. bacteriovorus* as a living antibiotic in medical and other applications. The authors also propose the use of optimal foraging theory in future modeling efforts to investigate the importance of spatially structured environments on predator characteristics such as the search for prey.

## Author contributions

All of the Research Topic editors, contributed to this editorial and approved it for publication.

